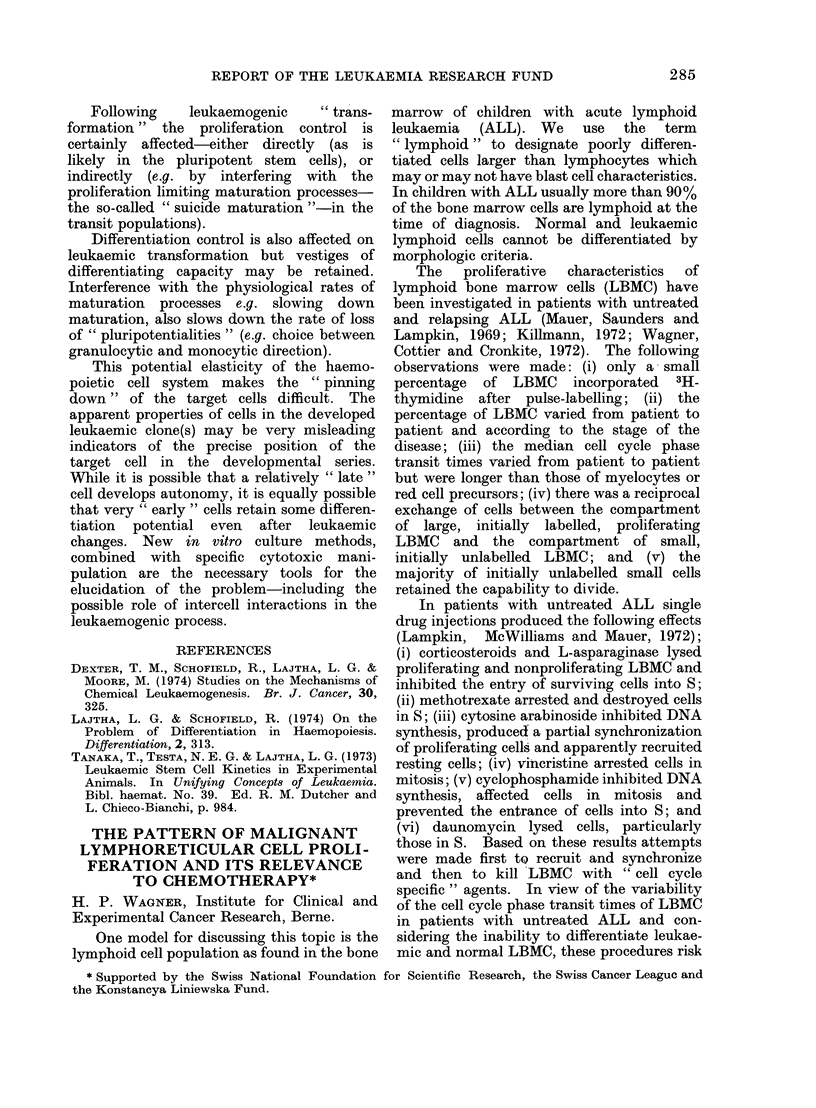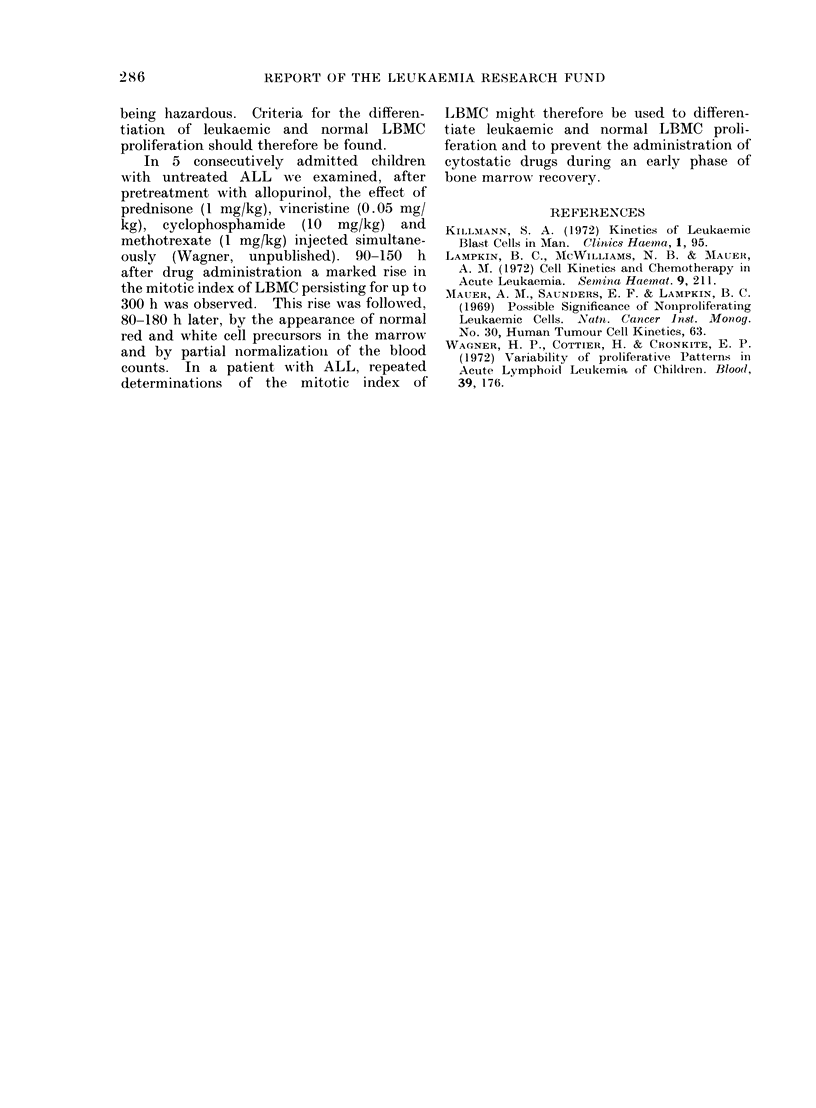# Proceedings: The pattern of malignant lymphoreticular cell proliferation and its relevance to chemotherapy.

**DOI:** 10.1038/bjc.1975.223

**Published:** 1975-08

**Authors:** H. P. Wagner


### THE PATTERN OF MALIGNANT LYMPHORETICULAR CELL PROLIFERATION AND ITS RELEVANCE TO CHEMOTHERAPY*

H. P. WAGNER, Institute for Clinical and Experimental Cancer Research, Berne.

One model for discussing this topic is the lymphoid cell population as found in the bone marrow of children with acute lymphoid leukaemia (ALL). We use the term "lymphoid" to designate poorly differentiated cells larger than lymphocytes which may or may not have blast cell characteristics. In children with ALL usually more than 90% of the bone marrow cells are lymphoid at the time of diagnosis. Normal and leukaemic lymphoid cells cannot be differentiated by morphologic criteria.

The proliferative characteristics of lymphoid bone marrow cells (LBMC) have been investigated in patients with untreated and relapsing ALL (Mauer, Saunders and Lampkin, 1969; Killmann, 1972; Wagner, Cottier and Cronkite, 1972). The following observations were made: (i) only a small percentage of LBMC incorporated $^3$H-thymidine after pulse-labelling; (ii) the percentage of LBMC varied from patient to patient and according to the stage of the disease; (iii) the median cell cycle phase transit times varied from patient to patient but were longer than those of myelocytes or red cell precursors; (iv) there was a reciprocal exchange of cells between the compartment of large, initially labelled, proliferating LBMC and the compartment of small, initially unlabelled LBMC; and (v) the majority of initially unlabelled small cells retained the capability to divide.

In patients with untreated ALL single drug injections produced the following effects (Lampkin, McWilliams and Mauer, 1972); (i) corticosteroids and L-asparaginase lysed proliferating and nonproliferating LBMC and inhibited the entry of surviving cells into S; (ii) methotrexate arrested and destroyed cells in S; (iii) cytosine arabinoside inhibited DNA synthesis, produced a partial synchronization of proliferating cells and apparently recruited resting cells; (iv) vincristine arrested cells in mitosis; (v) cyclophosphamide inhibited DNA synthesis, affected cells in mitosis and prevented the entrance of cells into S; and (vi) daunomycin lysed cells, particularly those in S. Based on these results attempts were made first to recruit and synchronize and then to kill LBMC with "cell cycle specific" agents. In view of the variability of the cell cycle phase transit times of LBMC in patients with untreated ALL and considering the inability to differentiate leukaemic and normal LBMC, these procedures risk

* Supported by the Swiss National Foundation for Scientific Research, the Swiss Cancer League and the Konstancya Liniewska Fund.

being hazardous. Criteria for the differentiation of leukaemic and normal LBMC proliferation should therefore be found.

In 5 consecutively admitted children with untreated ALL we examined, after pretreatment with allopurinol, the effect of prednisone (1 mg/kg), vincristine (0.05 mg/kg), cyclophosphamide (10 mg/kg) and methotrexate (1 mg/kg) injected simultaneously (Wagner, unpublished). 90–150 h after drug administration a marked rise in the mitotic index of LBMC persisting for up to 300 h was observed. This rise was followed, 80–180 h later, by the appearance of normal red and white cell precursors in the marrow and by partial normalization of the blood counts. In a patient with ALL, repeated determinations of the mitotic index of LBMC might therefore be used to differentiate leukaemic and normal LBMC proliferation and to prevent the administration of cytostatic drugs during an early phase of bone marrow recovery.

REFERENCES

KILLMANN, S. A. (1972) Kinetics of Leukaemic Blast Cells in Man. *Clinics Haema*, **1**, 95.

LAMPKIN, B. C., MCWILLIAMS, N. B. & MAUER, A. M. (1972) Cell Kinetics and Chemotherapy in Acute Leukaemia. *Semina Haemat.* **9**, 211.

MAUER, A. M., SAUNDERS, E. F. & LAMPKIN, B. C. (1969) Possible Significance of Nonproliferating Leukaemic Cells. *Natn. Cancer Inst. Monog.* No. 30, Human Tumour Cell Kinetics, 63.

WAGNER, H. P., COTTIER, H. & CRONKITE, E. P. (1972) Variability of proliferative Patterns in Acute Lymphoid Leukemia of Children. *Blood*, **39**, 176.